# Liraglutide Suppresses the Plasma Levels of Active and Des-Acyl Ghrelin Independently of Active Glucagon-Like Peptide-1 Levels in Mice

**DOI:** 10.1155/2013/184753

**Published:** 2013-08-13

**Authors:** Katsunori Nonogaki, Marina Suzuki

**Affiliations:** Department of Lifestyle Medicine, Translational Research Center, Tohoku University Hospital, 1-1 Seiryo-machi, Aoba-ku, Sendai, Miyagi 980-8574, Japan

## Abstract

Glucagon-like peptide-1 (GLP-1), an insulinotropic gastrointestinal peptide that is primarily produced by intestinal endocrine L-cells, stimulates satiety. Ghrelin, a hormone that is produced predominantly by the stomach stimulates hunger. There are two forms of ghrelin: active ghrelin and inactive des-acyl ghrelin. After depriving mice of food for 24 h, we demonstrated that the systemic administration of liraglutide (100 **μ**g/kg), a human GLP-1 analog that binds to the GLP-1 receptor, increased (1.4-fold) the plasma levels of active GLP-1 and suppressed the plasma levels of active and des-acyl ghrelin after 1 h. Despite the elevated plasma levels of active GLP-1 (11-fold), liraglutide had no effect on the plasma levels of active or des-acyl ghrelin after 12 h. These findings demonstrated that liraglutide suppresses the plasma levels of active and des-acyl ghrelin independently of active GLP-1 levels in fasted mice, suggesting a novel *in vivo* biological effect of liraglutide beyond regulating plasma GLP-1.

## 1. Introduction

Hunger is stimulated by ghrelin, a hormone that is primarily produced by the P/D1 cells that line the fundus of the stomach [1]. Plasma ghrelin levels increase during fasting and decrease after ingesting glucose or lipids but not protein [1]. The efferent vagus nerve contributes to the fasting-induced increase in ghrelin secretion [1, 2]. The ghrelin that is secreted in the stomach stimulates the afferent vagus nerve and promotes food intake [1]. Ghrelin exists in both inactive (des-acyl ghrelin) and active forms. Fasting increases both forms of ghrelin compared with the fed state. Hyperphagia and obesity decrease the plasma levels of des-acyl ghrelin but not of active ghrelin [1].

Satiety is stimulated by glucagon-like peptide-1 (GLP-1), an incretin hormone that is released from intestinal L-cells in response to nutrient ingestion [3–5]. The GLP-1 receptors (GLP-1Rs) are expressed in the central nervous system (CNS) and the afferent vagal nerve terminals and contribute to the anorexic effect of GLP-1 [3–6]. GLP-1 potentiates glucose-dependent insulin secretion by activating the GLP-1Rs that are expressed on pancreatic islet *β* cells [3–5]. GLP-1 secretion increases after ingesting glucose and lipids but not protein [6]. In the isolated rat stomach, GLP-1 has been reported to suppress ghrelin release [7]. In addition, GLP-1 suppresses plasma ghrelin levels in humans via insulin secretion in the late postprandial period [8].

Once GLP-1 is released from the L cells into the bloodstream, it is rapidly degraded from its active form (7–36) to an inactive, N-terminally truncated form (9–36) by dipeptidyl peptidase-4 (DPP-4) [3–5]. Liraglutide, a human GLP-1 analog, is a novel, long-acting GLP-1 derivative that is resistant to DPP-4 [3, 4, 9]. Its prolonged effects result from the substitution of Lys for Arg34 and the addition of a glutamic acid and a 16C fatty acid chain to the Lys26 residue of native GLP-1 [4, 9]. The effects of liraglutide on the plasma levels of active GLP-1, active ghrelin, and des-acyl ghrelin in fasted mice have not yet been evaluated. 

To determine the effects of liraglutide on the plasma levels of active GLP-1 and active and des-acyl ghrelin *in vivo*, we treated mice that were deprived of food for 24 h with an intraperitoneal injection of liraglutide.

To determine whether the effects of liraglutide on the plasma levels of active and des-acyl ghrelin resulted from the increased plasma levels of active GLP-1 *in vivo*, we measured the plasma levels of active GLP-1 and active and des-acyl ghrelin in mice 12 h after an intraperitoneal injection of liraglutide. 

## 2. Materials and Methods

### 2.1. Mice

Four-week-old male C57BL/6J mice were purchased from Japan CLEA. The mice were individually housed in cages with free access to water and chow pellets in a light- and temperature-controlled environment (12 h on/12 h off, lights on at 08:00 and lights off at 20:00; 20–22°C). The animals were acclimatized to the laboratory environment for 1 week before the experiment. 

In the first experiment, 5-week-old male C57BL/6J mice were deprived of food for 24 h and were then intraperitoneally injected with saline or liraglutide (100 *μ*g/kg). The animals were not fed chow pellets after being treated. Sixty minutes later, the animals were decapitated, and the blood was collected for the measurements of active GLP-1, active ghrelin, and des-acyl ghrelin.

In the second experiment, 5-week-old male C57BL/6J mice were intraperitoneally injected with saline or liraglutide (100 *μ*g/kg). The animals were not fed chow pellets after being treated. Twelve hours later, the animals were decapitated, and the blood was collected for the measurements of active GLP-1, active ghrelin, and des-acyl ghrelin.

The whole blood was mixed with EDTA-2Na (2 mg/mL) and aprotinin (500 kIU/mL) to determine the plasma levels of GLP-1 and active and des-acyl ghrelin. The dose of liraglutide (100 *μ*g/kg) was selected based on evidence that liraglutide induces hypophagia [10]. Liraglutide was a kind gift from Novo Nordisk, Japan. The drugs were dissolved in 0.2 mL 0.9% saline. 

The animal studies were conducted in accordance with the institutional guidelines for animal experiments at the Tohoku University Graduate School of Medicine. 

### 2.2. Plasma Ghrelin and GLP-1 Assay

The plasma levels of active and des-acyl ghrelin were measured by ELISA (Murine active ghrelin and des-acyl ghrelin ELISA kits; Mitsubishi Kagaku Iatron Inc., Tokyo, Japan). For the active ghrelin ELISA, 1 N hydrogen chloride was added to the samples immediately after the plasma separation to achieve a final concentration of 0.1 N, as described previously [11]. The plasma active GLP-1 levels were measured by ELISA (mouse active GLP-1 ELISA kit; Shibayagi Inc., Gunma, Japan) [12, 13]. 

### 2.3. Statistical Methods

Data are presented as the mean ± SEM (*n* = 6). The comparisons between two groups were performed with Student's *t*-test. A *P* value of less than 0.05 was considered to be statistically significant.

## 3. Results and Discussion

### 3.1. The Effects of Liraglutide on the Plasma Levels of Active GLP-1, Active Ghrelin, and Des-Acyl Ghrelin in Fasted Animals

**Figure 1:**
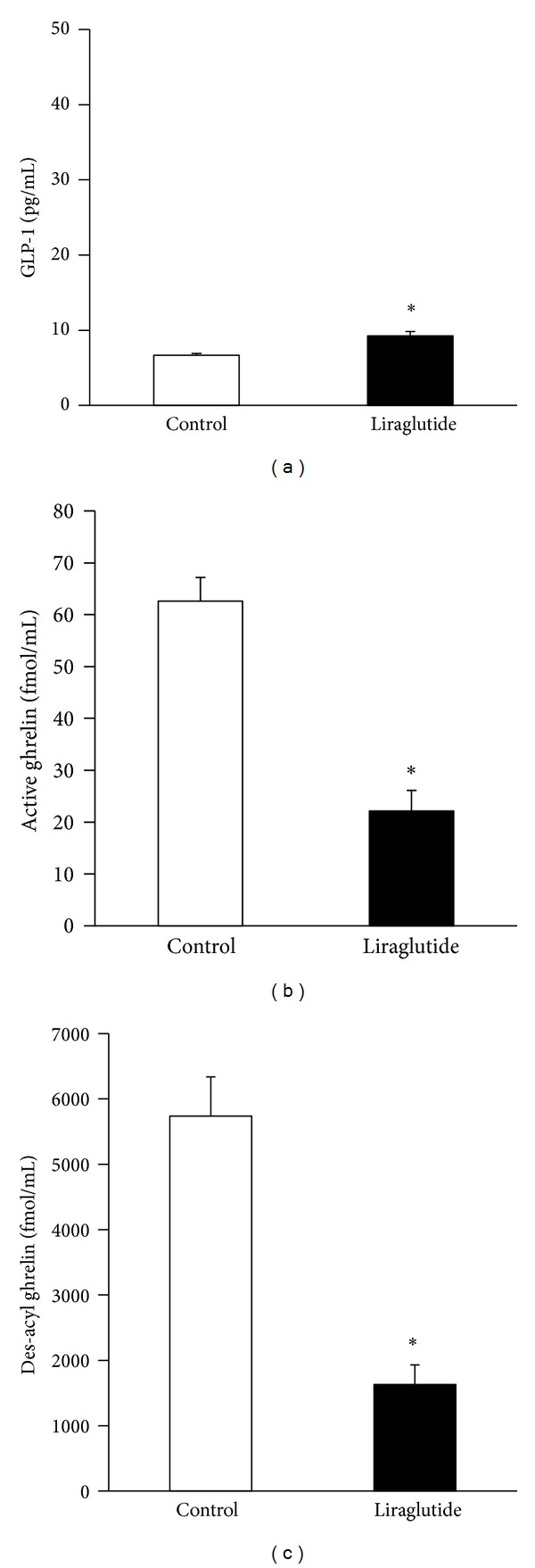
The effects of an intraperitoneal injection of liraglutide (100 *μ*g/kg) or saline on the plasma levels of active GLP-1 (a), active ghrelin (b), and des-acyl ghrelin (c) in mice fasted for 24 h are presented. The plasma levels of active GLP-1, active ghrelin, and des-acyl ghrelin were determined 1 h after liraglutide treatment, which was administered following 24 h of food deprivation, as described in the Materials and Methods section. The data are presented as the mean ± SEM (*n* = 6 for each group). *P < 0.05.

In mice that were fasted for 24 h, systemic liraglutide (100 *μ*g/kg) treatment significantly increased the plasma concentration of GLP-1 (1.4-fold) at 1 h compared with the saline control (Figure 1(a)). In addition, liraglutide (100 *μ*g/kg) significantly decreased the plasma levels of active (0.35-fold) and des-acyl (0.28-fold) ghrelin (Figures 1(b) and 1(c)). These findings suggested that liraglutide acutely suppressed the plasma levels of active and des-acyl ghrelin, which wasassociated with a small increase in circulating GLP-1in mice that were fasted for 24 h. 

### 3.2. The Effects of Liraglutide after 12 h on the Plasma Levels of Active GLP-1, Active Ghrelin, and Des-Acyl Ghrelin

**Figure 2:**
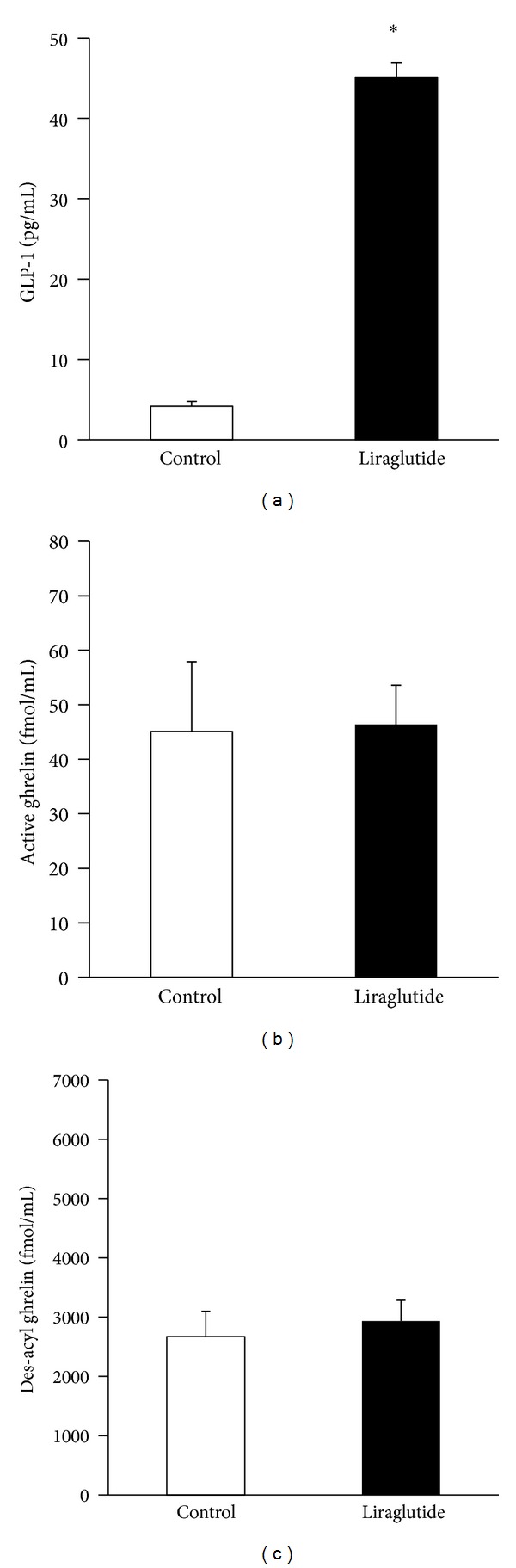
The effects of an intraperitoneal injection of liraglutide (100 *μ*g/kg) or saline on the plasma levels of active GLP-1 (a), active ghrelin (b), and des-acyl ghrelin (c) in mice are described. The plasma concentrations of active GLP-1, active ghrelin, and des-acyl ghrelin were determined 12 h after liraglutide treatment, as described in the Materials and Methods section. The data are presented as the mean ± SEM (*n* = 6 for each group). *P < 0.05.

In mice, the intraperitoneal injection of liraglutide (100 *μ*g/kg) increased the plasma level of active GLP-1 (11-fold) at 12 h compared with the saline control (Figure 2(a)). The intraperitoneal injection of liraglutide (100 *μ*g/kg) did not significantly affect the plasma levels of active or des-acyl ghrelin at 12 h (Figures 2(b) and 2(c)). These findings suggested that despite the remarkable elevated levels of active GLP-1, systemic liraglutide did not affect the plasma levels of active or des-acyl ghrelin in mice.

Liraglutide is 97% homologous to human GLP-1. The additional 16-carbon fatty acid chain on liraglutide noncovalently binds to albumin, which allows for absorption at the injection site and shields the molecule from degradation by DPP-4, thus protecting its activity [14]. Liraglutide has a half-life of thirteen hours [14]; therefore, the increased levels of active GLP-1 might result from exogenous human GLP-1 or a combination of exogenous human GLP-1 and endogenous murine active GLP-1. The present study demonstrated that the liraglutide-induced suppression of the plasma levels of active and des-acyl ghrelin was not related to the increased plasma level of active GLP-1 in the fasted mice. Because the efferent vagus nerve contributes to the fasting-induced increase in ghrelin secretion [2], liraglutide likely suppresses efferent vagus nerve-mediated ghrelin secretion in the fasted state. 

There are at least two potential neural mechanisms by which liraglutide could suppress the plasma levels of active and des-acyl ghrelin. First, the central GLP-1Rs could regulate the liraglutide-induced suppression of efferent vagal nerve-mediated ghrelin secretion because central GLP-1R stimulation with exendin-4, another long-lasting GLP-1R agonist, moderates the parasympathetic regulation of the heart, leading to an increased heart rate [15]. Second, the GLP-1Rs in the afferent vagal nerve terminals could contribute to the liraglutide-mediated suppression of plasma active and des-acyl ghrelin levels because liraglutide stimulates the vagal afferents and the CNS, leading to satiety [16]. GLP-1Rs are expressed on afferent vagal nerve terminals in the gastrointestinal and hepatoportal systems and on pancreatic *β* cells [3, 5]. The afferent vagal nerve enters the NST, which projects into hypothalamic structures that are involved in appetite regulation [17, 18]. GLP-1Rs that are expressed on the vagal afferent fibers of the gastrointestinal tract may contribute to GLP-1-induced satiety, but the common hepatic branch of the vagus nerve may not be required for this effect [19]. GLP-1 has been reported to suppress plasma ghrelin levels via insulin secretion in the late postprandial period [8]. Because liraglutide does not increase insulin secretion in the fasted state, insulin is not likely to contribute to the liraglutide-mediated suppression of fasting plasma ghrelin levels. Further studies are necessary to determine the mechanisms by which liraglutide suppresses the plasma levels of active and des-acyl ghrelin. 

## 4. Conclusions

These data suggest that liraglutide suppresses the plasma levels of active and des-acyl ghrelin independently of the plasma level of active GLP-1 in fasted mice. To the best of our knowledge, this is the first report of this novel biological effect of liraglutide and the effect of liraglutide on the plasma concentration of active GLP-1.
